# Neurofibroma involving obturator nerve mimicking an adnexal mass: a rare case report and PRISMA-driven systematic review

**DOI:** 10.1186/s13048-018-0386-z

**Published:** 2018-02-09

**Authors:** Wei-Ting Chao, Chia-Hao Liu, Yi-Jen Chen, Hua-Hsi Wu, Chi-Mu Chuang, Peng-Hui Wang

**Affiliations:** 1 Faculty of Medicine, College of Medicine, Fu-Jen Catholic University, Taipei, Taiwan; 2 Faculty of Medicine, School of Medicine, National Yang-Ming University, Taipei, Taiwan; 3 Section of Gynecologic Oncology, Department of Obstetrics and Gynecology, Taipei Veterans General Hospital, No. 201, Section 2, Shih-Pai Road, Taipei, 112 Taiwan; 4 Department of Midwifery and Women Health Care, National Taipei University of Nursing and Health Sciences, Taipei, Taiwan; 5 Department of Medical Research, China Medical University Hospital, Taichung, Taiwan

**Keywords:** Neurofibroma, Ovarian cancer, Retroperitoneal, Solitary

## Abstract

**Background:**

Pelvic masses are a common gynecologic problem, and majority of them are diagnosed as ovarian tumors finally. Sometimes, it is hard to distinguish the origin of these pelvic masses. The following case is a solitary neurofibroma arising from the right-side obturator nerve, which was impressed as a right-side ovarian tumor initially. We reported this case, and also performed a PRISMA-driven systematic review to summary the similar cases in the literature. This review includes image, molecular and pathological findings and outcome of neurofibroma.

**Case presentation:**

A 33-year-old woman with a regular menstrual period denied any symptoms or signs. During her physical check-up, image examination revealed a right-side heterogeneous pelvic mass; it was suggestive of a complex of right-side ovarian tumor. A provisional diagnosis of retroperitoneal pelvic mass, probably a benign ovarian tumor, was made.

Excision of the right-side pelvic mass was performed. We sent the specimens for frozen pathology, which indicated neurofibroma and lipomatous tumor and that the possibility of liposarcoma cannot be excluded. A segment of the obturator nerve was attached to the tumor and was severed. A right-side obturator nerve tear during tumor excision was observed, and a neurosurgeon was consulted for obturator nerve grafting and repair. The patient complained of mild weakness and paresthesia affecting the right leg, and we consulted a rehabilitation doctor for neuron injury. The patient’s recovery was uneventful, and she was discharged eight days after the drain was removed. Further rehabilitation treatment was arranged.

**Conclusion:**

A neurofibroma is an uncommon pelvic retroperitoneal tumor, and it can be misdiagnosed as an adnexal mass. To our knowledge, this is a rare case of a solitary neurofibroma arising from the obturator nerve. It usually does not have any neurological deficit. We present this case to demonstrate that pelvic neurofibroma can be mistaken for an adnexal mass. This fact should be borne in mind during the diagnosis process.

## Background

A neurofibroma is a benign soft tissue tumor arising from Schwann cells. Neurofibromas have been classically associated with neurofibromatosis type I (NF-1, Von Recklinghausen’s disease). They are found in diverse anatomical locations but seldom in the retroperitoneal location [1]. We report this case of a pelvic neurofibroma in a 33-year-old woman, which mimicked an adnexal mass on presentation. A systematic review was conducted to identify published relevant reports.

### Case presentation

**Fig. 1:**
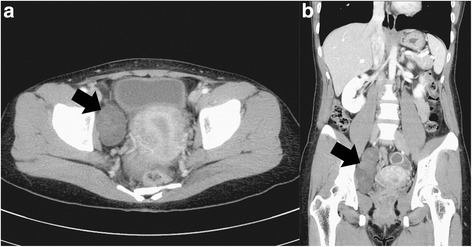
Computed tomography (**a**) axial and (**b**) sagittal view showing a right-side heterogeneous pelvic mass posterior to the urinary bladder pushing the uterus to the left side

A 33-year-old woman (gravida 2, para 0) with a regular menstrual period denied any symptoms or signs such as abdominal pain, dysmenorrhea, or menorrhagia. During a routine physical examination, ultrasonography revealed two right-side adnexal masses. One measuring 3.7 cm × 3.0 cm was suggestive of a right-side chocolate cyst. The other one measured 6.9 cm × 4.1 cm with vascularity on color-flow Doppler study. A computed tomography scan revealed a right-side heterogeneous pelvic mass posterior to the urinary bladder pushing the uterus to the left side (Fig. 1); it was suggestive of a complex of right-side ovarian tumor. A provisional diagnosis of retroperitoneal pelvic mass, probably a benign ovarian tumor, was made.

**Fig. 2:**
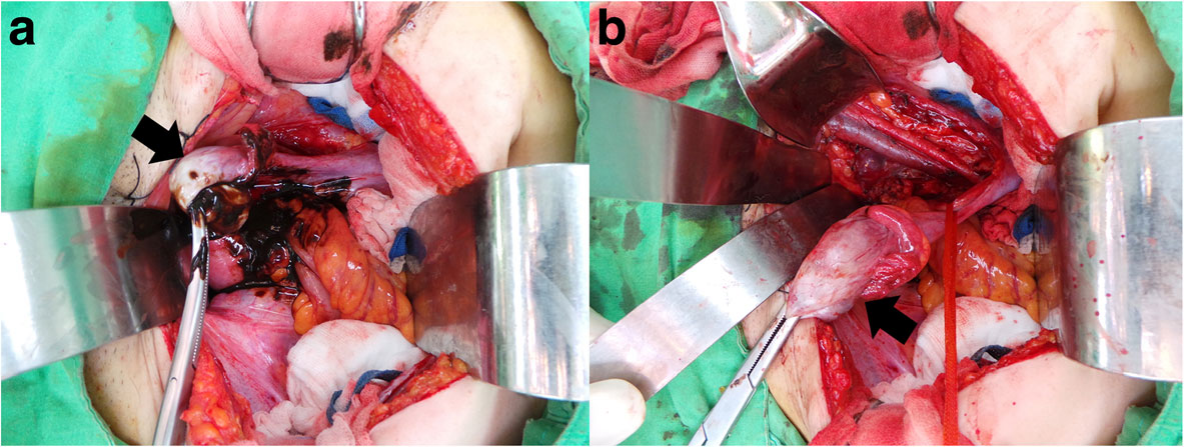
**a** A 3-cm right-side chocolate cyst. **b** Neurofibroma, 4 cm, attached to the right-side obturator nerve

Excision of the right-side pelvic mass and a right-side ovarian cystectomy were performed. The uterus was normal in size. One 3-cm right-side chocolate cyst (Fig. 2a) was visualized, and cystectomy was performed. The other 4-cm right-side pelvis mass (Fig. 2b) was identified, and tumor excision was performed. We sent the specimens for frozen pathology, which indicated (a) a spindle cell tumor and compatible with neurofibroma and (b) lipomatous tumor and that the possibility of liposarcoma cannot be excluded. A segment of the obturator nerve was attached to the tumor and was severed. A right-side obturator nerve tear during tumor excision was observed, and a neurosurgeon was consulted for obturator nerve grafting and repair. A Jackson–Pratt drain was kept on the right side of the abdomen, and the patient stood for the duration of the entire procedure—7 h and 30 min. The patient complained of mild weakness and paresthesia affecting the right leg, and we consulted a rehabilitation doctor for neuron injury. The patient’s recovery was uneventful, and the drain was removed on postoperative day 7, and she was discharged eight days after the drain was removed. Further rehabilitation treatment was arranged.

**Fig. 3:**
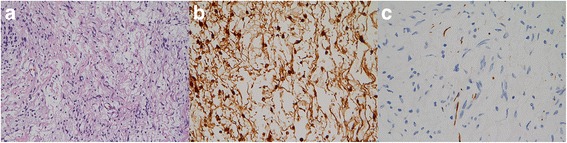
**a** Hematoxylin–eosin stain. Under the microscope, the neurofibroma is characterized by interlacing bundles of elongated cells with wavy, hyperchromatic nuclei. The tumor has a myxoid background with strands of collagen mimicking shredded carrots, × 200. **b** Stain of S-100. Part of the tumor cells is immunoreactive for S-100, × 400, (**c**) Stain of neurofilaments. Axons within the neurofibroma are demonstrated with neurofilaments, × 400

The surgical pathology report included a picture of the neurofibroma (Fig. 3a), which comprised oval to eel-like spindle nuclei on a background of generally wavy, arranged collagenous fibers and myxoid matrixes. Many areas contained schwannoma-like features, characterized by a biphasic pattern with compact areas of spindle cells alternating with loosely arranged foci containing collections of foamy histiocytes. The tumor was diffusely positive for S100 (Fig. 3b) and SOX-10. Several axons within the tumor were demonstrated with NF immunostain (Fig. 3c).

## Methods

### PRISMA-driven systematic review for solitary retroperitoneal neurofibroma

#### Search strategy of systemic reviews

An extensive literature review was performed in accordance with the guidelines published for PRISMA [2]. An expansive computerized systemic review of published reports, including meta-analysis, randomized controlled trials, cohort studies, and case reports, was performed by searching the following databases: PubMed, Ovid Medline, EMBASE, Cochrane Database of Systematic Reviews, Cochrane Central Register of Controlled Trials, and Google Scholar. The key search terms included “neurofibroma,” “retroperitoneal,” and “solitary”. The search was limited to human studies and full-text published in English from January 1960 to July 2017.

#### Screening and data extraction

The aforementioned searching strategy was completed by July 2017. Two independent reviewers (W.T.C. and C.M.C.) reviewed the relevance of all titles and abstracts identified from the computerized database. Full articles were further assessed when the abstracts appeared to meet the inclusion criteria. The reviewed data were obtained and entered onto an ad hoc standardized data entry form by each reviewer. We compared the inclusion data for the origin (continent/country), year of publication, case report text and discussion, length of recruitment period, and source of information.

#### Assessment of methodological quality

The quality of observational studies (e.g., database analysis, case report, and cohort study) was scored in accordance with the Newcastle–Ottawa Quality Assessment Scale, which ranges from 1 (*poor*) to 9 (*excellent*) [3]. Because no regular descriptions for this scale exist except for the lowest and highest scores, we classified studies with a total score equal to or greater than 7 as high-quality studies.

## Results

**Fig. 4:**
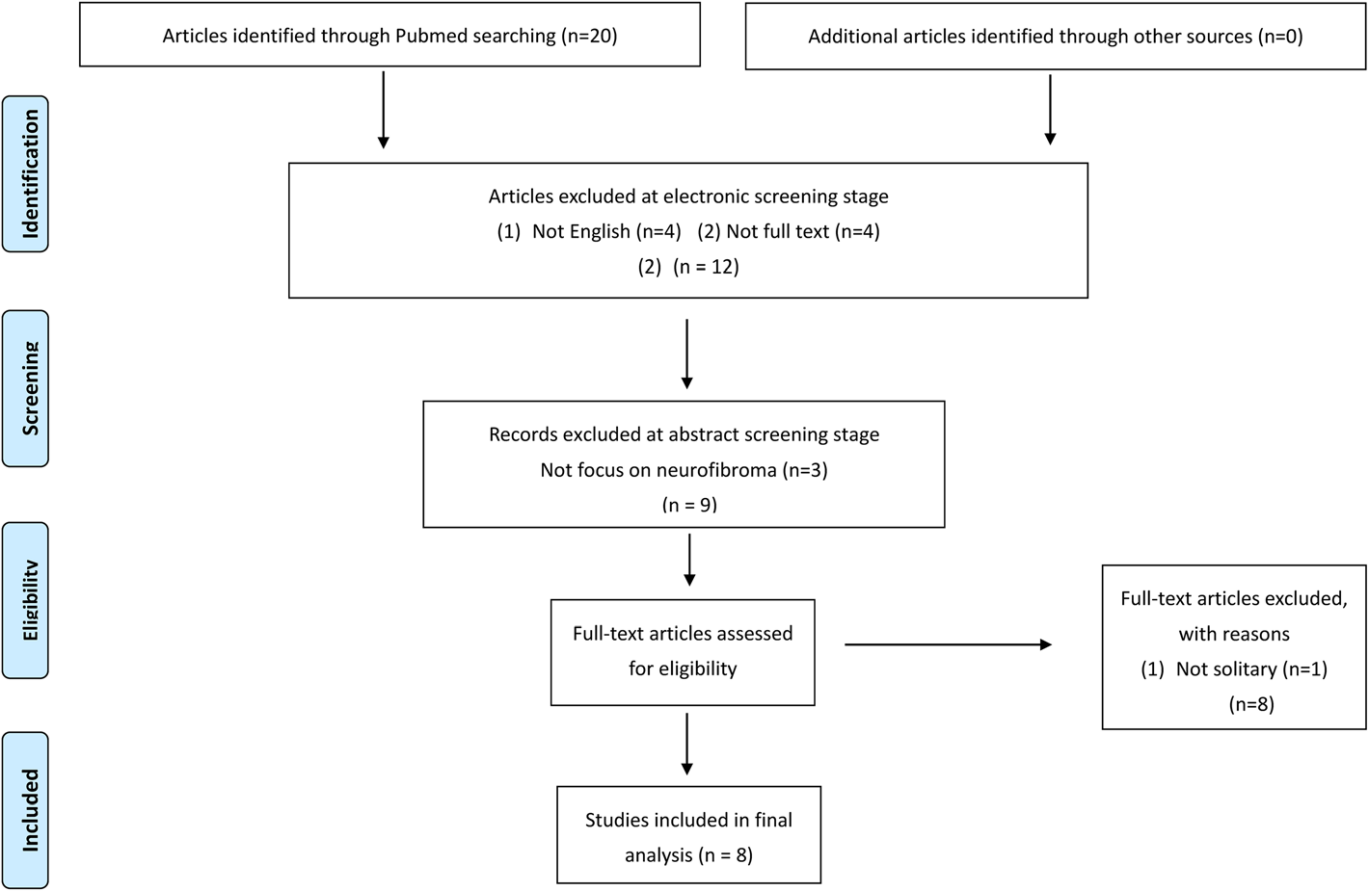
PRISMA flow diagram

Our search yielded 20 citations. After reviewing the title and abstract and reviewing the entire text, 12 were discarded as they did not meet the criteria proposed. In total, eight papers were identified for review, as illustrated in the PRISMA flow diagram (Fig. 4). The clinical characteristics of the eight included papers [4–11], associated with our case report, were summarized (total cases = 45) (Table 1).

**Table 1:** The included papers

Author	Year of publication	Gender	Age	Tumor location	Symptoms	Surgical intervention	Prognosis	Image study/Genetic analysis	Immunohistochemistry stain
Topsakal et al. [4]	2001	female	35	Right pre-sacral solitary neurofibroma	Bilateral sciatica radiating to the right inguinal and lumbar area for 2 years	Extra-peritoneal approach through a right J incision with complete resection	Uneventful without neurological deficit	Transvaginal ultrasound, Computed Tomography / Without gene of neurofibromatosis	Vimentin (+), Fibronectin(+), S-100(−), Cytokeratin(−), Desmin(−)
Kim et al. [5]	2013	female	72	Early gastric cancer with a 1.6-cm sized neurofibroma posterior to duodenum	Epigastric discomfort for 2 months	Laparoscopic assisted distal gastrectomy and retro-pancreatic nodal dissection	Uneventful without neurological deficit	Abdominal Computed Tomography / Without gene of neurofibromatosis	S-100 (+)
Dafford et al. [6]	2007	Total 38 patients	Total 38 patients	Pelvic retroperitoneal region	Pelvic pain, paresthesia or weakness, palpable pelvic mass	Total or subtotal resection	Moderate pain reduction	Magnetic resonance imaging, Computed Tomography / Without gene of neurofibromatosis	S-100 (+)
Shen et al. [7]	2016	female	45	Giant neurofibroma involving the pelvic cavity, retroperitoneal space and right buttock	Compressive displacement of abdominal and pelvic organs	En-bloc abdominopelvic tumor removal	Uneventful without neurological deficit	Computed Tomography / Not mention	Vimentin (+), CD-34(+), S-100(−), smooth muscle actin(−), desmin(−), cytokeratin(−)
Ishikawa et al. [8]	1989	female	56	Retro-vesical space	Dysuria for 2 years, traction pain in the left lower limb	Exploratory laparotomy	Uneventful without neurological deficit	Computed Tomography / Not mention	S-100 (+), neuron specific enolase(NSE) (+)
Hunter et al. [9]	1988	female	34	Pulsatile mass at the right pelvic side-wall	Right lower pelvic pain with sharp radiation into the right lower extremity for 3 years	Total tumor excision, right side J incision with extra-peritoneal approach	Loss of range of motion in the right foot, receiving physical therapy and improvement after 7 month of surgery	Intravenous pyelogram, cystoscopy / Not mention	Not mention
Cowles et al. [10]	1987	female	25	Soft mass extending from pubo-coccygeus muscle and para-vaginal space	Mild pelvic discomfort for one year exacerbated by prolong sitting	Surgical exploration and total excision	Uneventful without neurological deficit	Not mention / Not mention	Not mention
Gupta et al. [11]	2015	female	51	Over the left adrenal gland	Upper abdominal pain for 2 years	Laparoscopic left adrenalectomy	Uneventful without neurological deficit	Computed Tomography / Without gene of neurofibromatosis	S-100 (+)

Of these eight citations, the gender was female in seven cases and unknown in thirty-eight cases. Age varied from young to old. Tumor location was focused on the retroperitoneal space, presacral space, and pelvic cavity, and symptoms were dependent on the location of organic compression. Surgical intervention was included in laparoscopic-assisted tumor excision (*n* = 2) or surgical exploratory total or subtotal tumor excision (*n* = 6). The prognosis in most cases (*n* = 7) was uneventful without neurological deficit but one was under physical therapy; it improved after 7 months of surgery. Computed tomography, magnetic resonance imaging, and ultrasound are screening imaging tools, but diagnosis depends on pathologic reports and immunochemistry staining. Genetic analysis of four citations showed no neurofibromatosis gene, and the other was not mentioned. Immunochemistry staining of four cases showed positive S-100, and that of two cases showed negative S-100.

## Discussion

The lifetime risk is approximately 5%–10% for women undergoing surgery for a suspected ovarian neoplasm [12]. An adnexal mass may be found in females of all ages; it is a common gynecologic problem. Borgfeldt et al. investigated 335 woman between 25 and 40 years old who underwent transvaginal ultrasound examinations. The prevalence of adnexal mass lesions determined through ultrasound examination was 7.8% [13]. We present the case of a pelvic neurofibroma in a 33-year-old woman, which mimicked an adnexal mass. Topsakal et al. presented a case who was initially considered to have a giant ovarian mass, but was referred to a neurosurgeon for a solitary giant neurofibroma [4].

Neurofibromatosis is hereditary, and the clinical presentation could vary in the bones, nervous system, eyes, skin, gastrointestinal tract, and other body parts [14]. It is classified into two types. NF-1 is an autosomal-dominant inheritable syndrome because of genetic mutations in the coding of the neurofibromin [7], which is characterized by peripheral manifestations. Neurofibromas are benign tumors of the peripheral nerves and are usually considered pathognomonic of neurofibromatosis type 1 (NF-1). NF-1 is a disease that results from spontaneous or familial transmitted mutations in the NF-1 gene located on chromosome 17q11.2. These mutations cause a loss-of-function in the protein neurofibromin, which typically functions as a tumor suppressor. However, diagnosis of NF-1 was depending on clinical symptoms and criteria for diagnosis was established by the NIH in 1988 and is listed in Table 2. Our patient was found to lack the typical signs of NF-1 with absence of Lisch nodules, cafe’-au-lait spots, optic gliomas, multiple other neurofibromas or axillary/inguinal freckling. Computed tomography did not showed other mass lesion of spinal cord or central nervous system. With clinical exclusion of NF-1, she was determined to have a solitary neurofibroma. Type II neurofibromatosis has alternative presentations of the central nervous system [14]. Solitary neurofibromas are solid and nonencapsulated tumors that tend to grow slowly in the skin of people aged 20–30 years and is present without sex differences. Neurofibromas rarely arise from other parts of the body [14]. Most patients with retroperitoneal pelvic neurofibroma have associated neurofibromatosis/Von Recklinghausen’s disease [1, 15, 16]. Our patient did not have any hereditary disorder. The present case of pelvic retroperitoneal neurofibroma indicates that these tumors can arise de novo with neither genetic disorder nor other particular organ involvement. A patient with a single neurofibroma represents a true sporadic case or carries the defective gene with only mild clinical presentation [17]. It may become malignant [15, 16]; however, malignant transformation was rare, except in the 4%–11% of cases associated with NF-1 [18].

**Table 2:** Gives NIH consensus guidelines for type I neurofibromatosis diagnostic criteria

NIH consensus guidelines: diagnostic criteria for neurofibromatosis I. Two or more of the following	
1. Six or more cafe’-au-lait macules that are (in greatest diameter) > 5 mm in pre-pubertal individuals > 15 mm in post-pubertal individuals	
2. Two or more neurofibromas of any type, or one plexiform neurofibroma	
3. Axillary/inguinal freckling	
4. Optic glioma	
5. Two or more Lisch nodules	
6. Distinctive osseous lesion (i.e. sphenoid dysplasia or thinning of long bone cortex with or without pseudoarthrosis)	
7. First degree relative with NF-1	

Patients with neurofibroma usually do not have any neurological symptoms [1]. These tumors, unlike other peripheral nerve tumors, may grow to a considerable size and occupy an unusual position, compressing the neighboring structures and causing severe pain. The most common symptom of retroperitoneal tumors is inadequately localized pain that may be present in the genitalia or lower extremities, often accompanied by numbness, tingling, and, occasionally, urinary symptoms [4]. Other studies have reported that the other clinical manifestations of solitary neurofibromas depend on their location. Shen et al. presented a case of a 45-year-old-female who underwent surgical intervention for a giant, retroperitoneal neurofibroma that compressed the visceral and genitoanal organs [7]. Topsakal et al. presented a 35-year-old woman with a solitary presacral neurofibroma without neurofibromatosis manifesting as bilateral chronic sciatica for 2 years [4] who was initially diagnosed with a giant right ovarian mass.

If a case has no cutaneous neurofibromatosis, diagnosis of pelvic neurofibroma is made only on histology of the excised specimen [1]. When a diagnosis of solitary neurofibroma is made, other subclinical forms of neurofibromatosis must be excluded. The mass must be confirmed as solitary [14]. Computed tomography is a useful preoperative diagnostic imaging tools. A neurofibroma arising from the presacral location may displace the uterus and rectum [4]. However, a neurofibroma arising from the obturator nerve did not displace the visceral organs and it made making the correct diagnosis—either ovarian mass or neural sheath tumor—more difficult. Systemic review of description of image study of solitary neurofibroma was also analyzed (Table 3). Computed tomography scan revealed hypodense lesion with intermediate contrast-enhancement, it was easily confused with benign ovarian tumor if the location of lesion was close to adnexa. Our patient did not receive Magnetic resonance imaging, however, it could differentiate ovarian tumor and neurofibroma. According to our systemic review, neurofibroma was high intensity with a well-circumscribed low intensity center or low intensity surrounding under T2-weighted image and T1-weighted image was intermediate intensity. We strongly advised patient to receive Magnetic resonance imaging for correct provisional diagnosis to differentiate.

**Table 3:** Description of image study

Author	Year of publication	Description of Image study	
		Computed Tomography	Magnetic resonance imaging
Topsakal et al. [4]	2001	Smooth-contoured hypodense lesion with intermediate contrast-enhancement	T1-weighted image: intermediate intensity T2-weighted image: high intensity with a well-circumscribed low intensity center. It was also partially enhanced with contrast-medium
Kim et al. [5]	2013	A well-defined, 1.6 cm sized ovoid retroperitoneal mass with intermediate contrast-enhancement	Not mention
Dafford et al. [6]	2007	Low attenuating and hypodense mass lesion	T2-weighted image: High signal intensity with a low intensity surrounding. Hypointense septations have also been reported
Shen et al. [7]	2016	Hypodense lesion and with partially contrast-enhancement	Not mention
Ishikawa et al. [8]	1989	Well encapsulated homogeneous and hypodense mass lesion	Not mention
Hunter et al. [9]	1988	Not mention	Not mention
Cowles et al. [10]	1987	Not mention	Not mention
Gupta et al. [11]	2015	Heterogeneously intermediate enhancing mass lesion with calcification	Not mention

Complete surgical resection is the only treatment for these tumors [14]. The neurofibroma can recur after surgical intervention [19], but a complete resection is preferred to prevent local recurrence and malignant transformation [4]. Malignant transformation and recurrence are unusual [14]. Nerve root sacrifice is often required to achieve total tumor excision, but resection does not always result in a postoperative neurological deficit [4]. Levy et al. analyzed 66 spinal neurofibromas and determined that nerve fibers involved in a neurofibroma can usually be resected. This suggests that these nerve roots retained no function and would not degenerate further [20].

The exact pathogenesis of solitary neurofibroma remains unclear and genetic studies of neurofibromas in the literature are scarce. Systemic review of genetic analysis of solitary neurofibroma was also analyzed (Table 4). Beert et al. identified a bi-allelic NF inactivation as the cause of solitary neurofibroma in a 13-year-old boy without other NF diagnostic criteria [21]. Bi-allelic inactivation leading to the development of neurofibroma seems to occur in embryonic cells, which have the potential of cells carrying a NF second-hit mutation. Jungmann et al. identified p.P733L mutation in exon 15 of the *KIT* gene in a 22-year-old man with solitary neurofibroma on the left flank [22]. Sawyer et al. revealed reciprocal translocation t(4;9)(q31;p22) in a 29-year-old woman with solitary neurofibroma on her left arm [23]. Sugiyama et al. presented abnormal gains in NF-1 gene (17q11.2) by the single nucleotide polymorphism identification method in a 33-year-old man with solitary epicranial neurofibroma [24]. Solitary neurofibromas in “clinically” non-NF-1 patients are benign tumors and the identification of genetic aberrations in these tumors are not expected to play a role in diagnosis. However, it was believed that genetic sporadic mutation or familial germline mutation were essential for pathogenesis and further genetic investigation combined with more case experience can provide more information of pathogenic mechanism.

**Table 4:** Genetic analysis

Author	Year of publication	Family history	Genetic analysis of mutation
Beert et al. [21]	2012	No	Insertion of chromosomal bands 1p36-p35 at 17q11.2, in 11 of 18 analyzed cells (Biallelic *NF1* inactivation).
Jungmann et al. [22]	2016	Yes	p.P733L mutation in exon 15 of the *KIT* gene, while wild-type sequences were found in *KIT* exons 8, 9, 11, 13, 14 and 17.
Sawyer et al. [23]	2004	Yes	Reciprocal translocation t(4;9)(q31;p22)
Sugiyama et al. [24]	2014	No	Abnormal gains in NF1 gene (17q11.2)

A solitary retroperitoneal neurofibroma is rare, and it can be misdiagnosed as an adnexal mass. A neurofibroma can be associated with NF-1; however, a solitary pelvic neurofibroma probably arises de novo without other organ involvement. It usually does not have any neurological deficit. Inadequately localized pain is the most common symptom, and the other clinical symptoms depend on the tumor location. Histology of the excised tumor specimen is the only diagnostic tool. Computed tomography could facilitate preoperative diagnosis. Complete surgical resection is the only treatment for such tumors—it is preferred because it prevents local recurrence and malignant transformation. We present this case to demonstrate that pelvic neurofibroma can be mistaken for an adnexal mass. This fact should be borne in mind during the diagnosis process.

## Abbreviations

NF: Neurofibromatosis
NF-1: Neurofibromatosis type I
*NIH*: National Institutes of Health
*PRISMA*: Preferred Reporting Items for *Systematic* Reviews and Meta-Analyses
*SOX-10*: SRY-related HMG-box-10
